# Repurposing a photosynthetic antenna protein as a super-resolution microscopy label

**DOI:** 10.1038/s41598-017-16834-z

**Published:** 2017-12-01

**Authors:** Samuel F. H. Barnett, Andrew Hitchcock, Amit K. Mandal, Cvetelin Vasilev, Jonathan M. Yuen, James Morby, Amanda A. Brindley, Dariusz M. Niedzwiedzki, Donald A. Bryant, Ashley J. Cadby, Dewey Holten, C. Neil Hunter

**Affiliations:** 1 Department of Molecular Biology and Biotechnology, Firth Court, Western Bank, Sheffield, S10 2TN UK; 2 Department of Chemistry, Washington University, St. Louis, MO 63130 USA; 3 Department of Biochemistry and Molecular Biology, The Pennsylvania State University, University Park, Pennsylvania, 16802 USA; 4 Department of Physics and Astronomy, Hicks Building, Hounsfield Road, Sheffield, S3 7RH UK

## Abstract

Techniques such as Stochastic Optical Reconstruction Microscopy (STORM) and Structured Illumination Microscopy (SIM) have increased the achievable resolution of optical imaging, but few fluorescent proteins are suitable for super-resolution microscopy, particularly in the far-red and near-infrared emission range. Here we demonstrate the applicability of CpcA, a subunit of the photosynthetic antenna complex in cyanobacteria, for STORM and SIM imaging. The periodicity and width of fabricated nanoarrays of CpcA, with a covalently attached phycoerythrobilin (PEB) or phycocyanobilin (PCB) chromophore, matched the lines in reconstructed STORM images. SIM and STORM reconstructions of *Escherichia coli* cells harbouring CpcA-labelled cytochrome *bd*_1_ ubiquinol oxidase in the cytoplasmic membrane show that CpcA-PEB and CpcA-PCB are suitable for super-resolution imaging *in vivo*. The stability, ease of production, small size and brightness of CpcA-PEB and CpcA-PCB demonstrate the potential of this largely unexplored protein family as novel probes for super-resolution microscopy.

## Introduction

Photosynthetic organisms have evolved to absorb and trap solar energy with high efficiency, and consequently they employ a diverse range of pigments, housed in a variety of protein and membrane environments. Extended light-harvesting networks comprising hundreds and even many thousands of pigments form antenna systems that increase both the spatial and spectral cross-section for light harvesting. Subsequently, absorbed energy migrates to specialised protein centres for transient storage as a series of charge separation events. One such light-harvesting antenna is the phycobilisome of cyanobacteria and some red algae, which funnels absorbed solar energy towards the Photosystem I and II reaction centre complexes^1^. The phycobilisome is a large multimeric, extramembranous protein complex that usually consists of a tricylindrical core of trimeric allophycocyanin. Peripheral rods of stacked hexameric phycocyanin subunits extend from the core, and in some circumstances these rods are extended with additional phycobiliproteins, either phycoerythrin or phycoerythrocyanin^1,2^. The light absorbing pigments in the phycobilisome, the phycobilins, are linear tetrapyrroles that are covalently bound to their cognate proteins. One such phycobiliprotein is the ~18-kDa CpcA, which naturally binds phycocyanobilin (PCB) but can also bind phycoerythrobilin (PEB), phycoviolobilin (PVB), phycourobilin (PUB) or phytochromobilin (PϕB) when appropriate enzymes are expressed heterologously in *Escherichia coli* ^3^.

The specialized light-harvesting and energy-transfer functions of phycobiliproteins have practical applications. These proteins form a range of stable, soluble, vividly coloured and highly fluorescent complexes used as fluorescent probes in epifluorescent imaging^4–6^. However, traditional optical imaging is fundamentally limited in terms of resolving power due to the wave-like nature of light, which results in the detected signal of a single molecule being spread over a much larger area than the molecule actually occupies. This is especially problematic for closely spaced molecules, which become indistinguishable.

There are several different techniques that overcome this problem and achieve sub-diffraction imaging. The most common method is localization microscopy (LM)^7,8^, which is further sub-divided into Stochastic Optical Reconstruction Microscopy (STORM) and Photoactivated Localization Microscopy (PALM). LM overcomes the diffraction limit by taking advantage of the blinking properties of certain fluorophores that switch between bright and dark states. When only a few molecules are actively emitting at any time they can be observed in isolation from all others as a series of discrete point spread functions, which reveal their location^9^. Switching subsets of the bright and dark populations creates a process by which each of the molecules can be imaged in isolation and a super-resolved image can be reconstructed. Other techniques of achieving sub-diffraction imaging include Structured Illumination Microscopy (SIM)^10,11^, which uses a high-frequency structured illumination that is scanned across the sample through at least three different angles, yielding a series of moiré patterns, to uniformly increase the resolution of the final image. Through this patterned approach SIM is able to double the resolution, or effective numerical aperture, of an optical microscope.

A typical LM experiment uses subsets of organic fluorophores and fluorescent proteins that exhibit the requisite blinking properties. Whilst organic fluorophores typically emit more photons per photoswitch than photoactivatable fluorescent proteins, leading to a higher localization precision, they have to be accurately targeted to the proteins of interest. Translational fusions with a fluorescent protein overcome difficulties with targeting that arise from staining with an external fluorophore, but their use in LM is limited because their brightness is generally lower than that for organic dyes^12^. Given the limitations of photoactivatable members of the green fluorescent protein (GFP) family, for example, it is therefore of interest to develop small, stable, bright, photo-switchable, fluorescent proteins for applications in LM.

Here, we show that the 17.6 kDa α-subunit of phycocyanin, CpcA, bearing either a PCB or PEB chromophore, represents a novel, photo-switchable probe for super-resolution microscopy. Photo-switching behaviour has been established both *in vitro* using surface-attached, nanopatterned CpcA-PCB and CpcA-PEB arrays and *in vivo* by using CpcA as a reporter of membrane protein distribution in the bacterial cytoplasmic membrane. We also demonstrate the potential for using CpcA-labelled systems for *in vivo* SIM imaging.

## Results

### Photophysical properties of CpcA-PCB and CpcA-PEB

**Figure 1:**
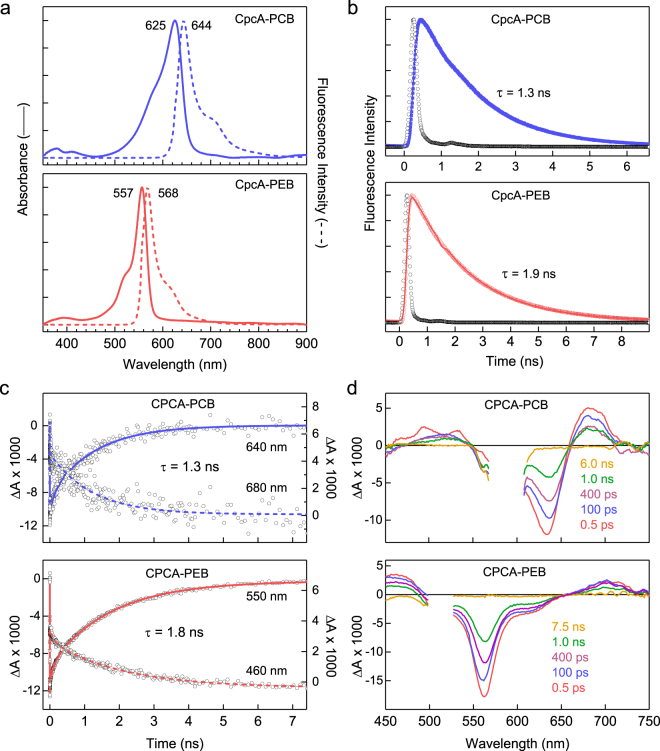
Photophysical analysis of purified CpcA-PCB and CpcA-PEB. (**a**) Room temperature absorption and fluorescence emission spectra, normalised for comparison. The same fluorescence spectra were observed for a number of different excitation wavelengths for CpcA-PCB (500, 525, 550, 572 and 625 nm) and CpcA-PEB (490, 510, 525 and 557 nm). (**b**) Fluorescence decay profiles (solid blue and red circles) and dual-exponential fits (solid blue or red lines) of CpcA-PCB using excitation at 582 nm and detection at 644 nm and CpcA-PEB using excitation at 530 nm and detection at 568 nm; the time constants indicated are of the dominant, longer component. The open black circles give the instrument response function, which is approximatly a Gaussian with a full width at half maximum of 200 ps. (**c**) Representative time profiles (circles) and fits for decay of ground state bleaching (solid lines) or excited state absorption (dashed lines) from the transient absorption data depicted in (**d**). (**d**) Time-resolved absorption difference spectra using 100-fs excitation flashes at 590 nm for CpcA-PCB or 510 nm for CpcA-PEB. The data in the region of each spectrum that contains scattered excitation light has been removed.

CpcA-PCB and CpcA-PEB were overproduced in *E. coli* and purified as described in Materials and Methods. Purified CpcA-PCB and CpcA-PEB proteins were analysed by steady-state and time-resolved absorption and fluorescence spectroscopy. Absorption and fluorescence emission spectra (Fig. 1A) revealed that these pigment-proteins occupy different but overlapping spectral regions, in total spanning 500–650 nm for absorption and 560–760 nm for emission. The singlet excited state lifetimes of CpcA-PCB and CpcA-PEB were measured by fluorescence decay (Fig. 1B) and transient absorption (Fig. 1C) studies. The average values for CpcA-PCB and CpcA-PEB are 1.3 and 1.9 ns, respectively. The fluorescence quantum yields were determined by relative and absolute measurements. The average values are 0.23 for CpcA-PCB and 0.67 for CpcA-PEB; both are lower than those reported previously (0.39 and 0.98)^3^ but have the same relative ordering. In order to ensure that the fluorescence spectra and quantum yields are not compromised by effects of aggregation, the spectra were measured for CpcA-PEB over a five-fold range of concentration (Supplementary Figure S1). This gave samples with an optical density (OD) at the 556 nm absorption peak ranging from 0.031 to 0.167 and the OD at the 510 nm excitation wavelength (to acquire fluorescence spectra) ranging from 0.009 to 0.046. The same absorption and fluorescence spectral profiles were obtained. Similarly, the integrated fluorescence intensities (when corrected for absorbance at the excitation wavelength) were the same. These findings indicate there are no significant effects of aggregation for such samples.

The transient absorption studies also allow measurement of the yield of intersystem crossing from the lowest singlet to triplet excited state, also known as the triplet yield. Representative time-resolved difference (ΔA = A_excited-state_ − A_ground-state_) spectra as a function of time after excitation with a 100 fs flash for CpcA-PCB are shown in Fig. 1D. In these spectra, positive-going features represent excited state absorption (e.g., S_1_ → S_2_, S_3_, etc.) and negative-going features reflect bleaching of a ground state absorption band (S_0_ → S_1_, S_2_, etc.) along with excited state emission (S_1_ → S_0_) stimulated by the white light probe pulse. The spectra for CpcA-PCB in Fig. 1D are dominated by bleaching of the ground state absorption band at ~640 nm due to formation of the lowest singlet excited state. This bleaching (and the rest of the absorption difference spectrum) decays to zero by 6 ns. This observation shows that the fluorescent lowest singlet excited state decays virtually completely to the ground state and thus that the yield of intersystem crossing to the lowest triplet excited state is virtually zero. The same conclusion is drawn for CpcA-PEB given that the bleaching of the ground state absorption band at ~570 nm for the lowest singlet excited state decays to zero by 7.5 ns (Fig. 1D). The transient absorption data depicted in Fig. 1 were acquired using excitation with 100 fs pulses having an energy of 1 μJ focused to 1 mm (2 × 10^14^ photons/cm^2^). This photon density was chosen to minimize singlet-singlet annihilation and other multiphoton effects that could compromise singlet decay characteristics and triplet yields. Therefore, studies on CpcA-PEB were undertaken using excitation energies of 0.25, 0.5, 1.0, 1.5 and 2.0 μJ/pulse. The same results − a triplet yield of essentially zero − was found in all cases. The main difference is that at the highest excitation energies, a fast (<10 ps) component to the excited state decay was observed, likely due to some singlet-singlet annihilation.

Thus, for both variants the yield of intersystem crossing is zero and the yield of internal conversion of the singlet excited state to the ground state can be obtained from the fluorescence yield. In particular, the internal-conversion yield is 1− 0.23 = 0.77 for CpcA-PCB and 1− 0.67 = 0.33 for CpcA-PEB. The finding that these proteins do not produce triplet excited states eliminates a mechanism for photo-instability in which long lived triplet states in the presence of O_2_ produce reactive oxygen species that could lead to degradation of the fluorescence probe (or other entities nearby). The photostability of CpcA-PEB appears to be somehow lower than that for CpcA-PCB in certain imaging studies (*see below*). Such a difference must then arise from processes initiated in the singlet excited state, such as photoinduced electron transfer (e.g. photooxidation) that would depend on the relative excited state energies and redox potentials of the two constructs.

The values for fluorescence lifetime and quantum yield are similar to those for variants of the enhanced yellow fluorescent protein (EYFP), for example, which are 3 ns and 0.76 respectively^13^. The extinction coefficients (M^−1^ cm^−1^) were calculated to be 100,265 M^−1^cm^−1^ at 624 nm for CpcA-PCB and 112,590 M^−1^cm^−1^ at 556 nm for CpcA-PEB. The overall brightness of these proteins is the product of the quantum yield and extinction coefficient, expressed in units of mM^−1^cm^−1^, which yields values of 23 and 75 for CpcA-PCB and CpcA-PEB, respectively, and compares well to EYFP, which has a brightness of 55.

### Photo-switching and STORM of nanopatterned arrays of CpcA-PCB and CpcA-PEB

To test the suitability of CpcA proteins as fluorophores suitable for STORM, we imaged nanopatterned arrays of the phycobiliproteins on a glass surface so the image to be reconstructed was known at the outset. The two types of CpcA were nanopatterned onto self-assembled monolayers on glass coverslips; these nanostructures have been well characterized by atomic force microscopy (AFM) and were used previously to study fluorescence lifetimes of photosynthetic complexes^14^. The samples were excited with either 514 nm (CpcA-PCB) or 532 nm (CpcA-PEB) lasers, which caused each of these pigmented proteins to undergo spontaneous photo-switching without the requirements of an activation laser. CpcA-PCB and CpcA-PEB settle into an on-off equilibrium with little evidence of photobleaching after an initial illumination period, a property required for photoactivatable fluorescent proteins.

**Figure 2:**
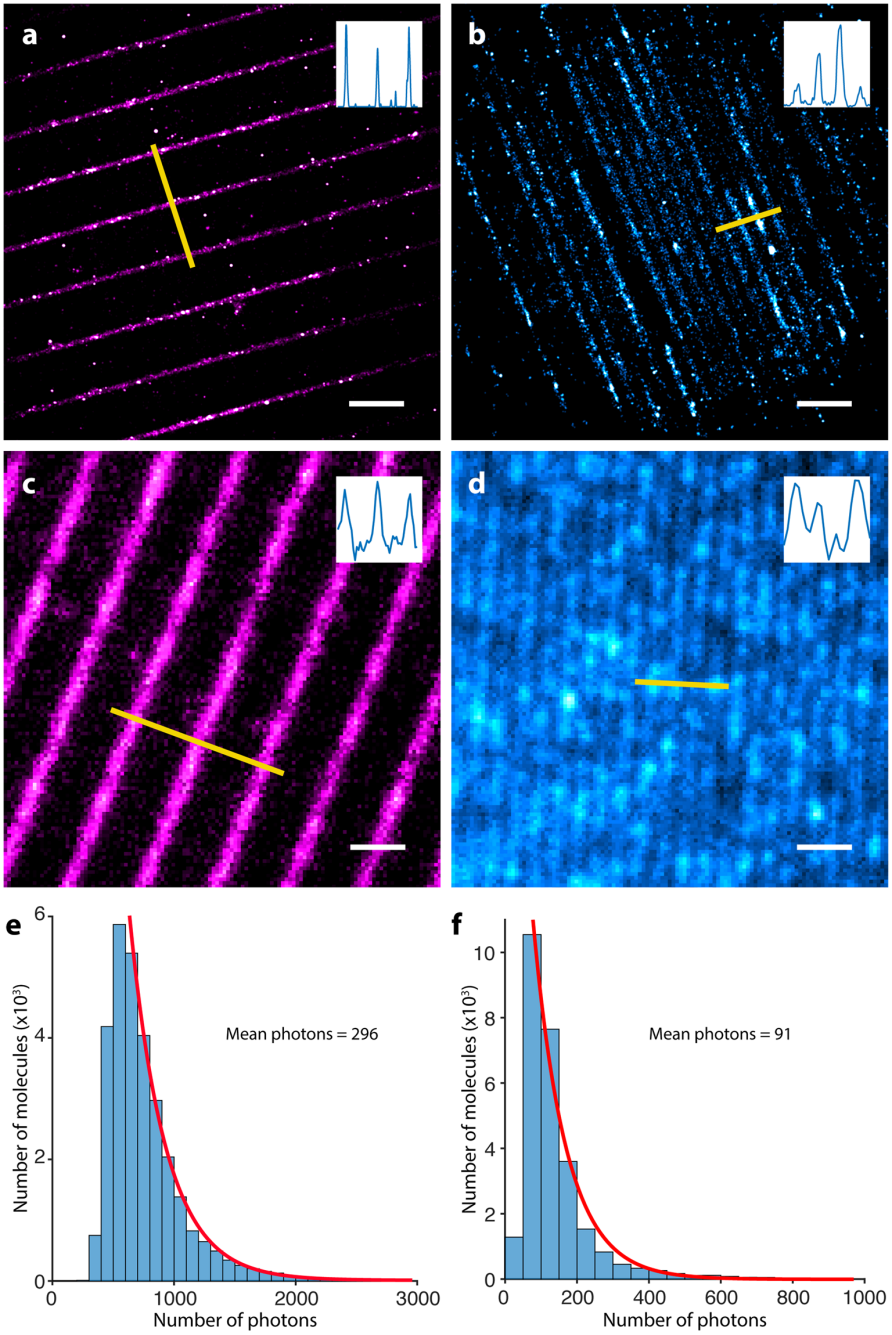
Reconstructions of STORM imaging of CpcA-PEB (**A**) and CpcA-PCB (**B**) nanopatterns. (**C**,**D**) Epi-fluorescent images of CpcA-PEB and CpcA-PCB nanopatterns respectively. Inset in the top-right corner of each image is the orthogonal line profile shown in yellow. Scale bar in bottom right of each image is 2 μm. (**E**,**F**) Mean number of photons as calculated by a single-term exponential fit of the frequency against photon number data of CpcA-PEB and CpcA-PCB respectively.

CpcA-PEB nanopatterns had been fabricated with 200 nm wide lines and 2 µm periodicity. The reconstruction process was performed with ThunderSTORM^15^, and the resulting image consisting of 66,780 events is shown in Fig. 2A. Analysis of the reconstructions by Fourier transform yielded a periodicity of 1.95 µm, in good agreement with the template used for lithography, and a linewidth (full-width half-maximum) of 200 nm. CpcA-PCB nanopatterns were created using a template with lines 303 nm wide and with a 606 nm periodicity. During imaging, both varieties of CpcA exhibited low densities of emissive species that allowed single molecule fitting with a mean 0.0109 events per µm^2^ per frame for CpcA-PEB and 0.004 events per µm^2^ per frame for CpcA-PCB. A reconstruction of the data obtained from recording 27,392 blinking events is shown in Fig. 2B; Fourier transform analysis yields a periodicity of 610 nm. These measurements are consistent with the dimensions from AFM topographs (Supplementary Figure S2), which gave linewidths of 303 nm. The linewidths obtained from reconstructions in Fig. 2A,B are compared with the broader linewidths from the epifluorescence images in Fig. 2C,D.

As is common with fluorescent proteins, the photon yield of each protein is lower than conventional dyes used in single molecule experiments. Single exponential fits of the photon counts for each protein (Fig. 2E,F) give a mean number of photons for each blink as 296 (CpcA-PEB) and 91 (CpcA-PCB).

Investigation of the switching properties of CpcA was performed on sparse protein attached to a glass coverslip such that the density allowed individual molecules to be observed. The on-switching rate of the proteins were investigated by finding the time-period between successive activations and fitting to an exponential function. The off-rate was found in a similar process but involved the emission lifetime of an individual event. This yielded on-off ratios of 2.67 × 10^−4^ and 1.71 × 10^−4^ for CpcA-PEB and CpcA-PCB respectively (see Supplementary Figure S3). The contrast ratios of the dark and light states for CpcA-PEB and CpcA-PCB proteins are demonstrated in Supplementary Figure S3 and show the dark state of CpcA-PCB to be slightly emissive compared to the bright state; however, CpcA-PEB shows zero emission in the dark state. The mean localisation uncertainty for CpcA-PEB and CpcA-PCB were calculated as part of the reconstruction process by ThunderSTORM and yielded values of 14 nm and 20 nm respectively (see Supplementary Figure S4).

### *In vivo* STORM imaging

**Figure 3:**
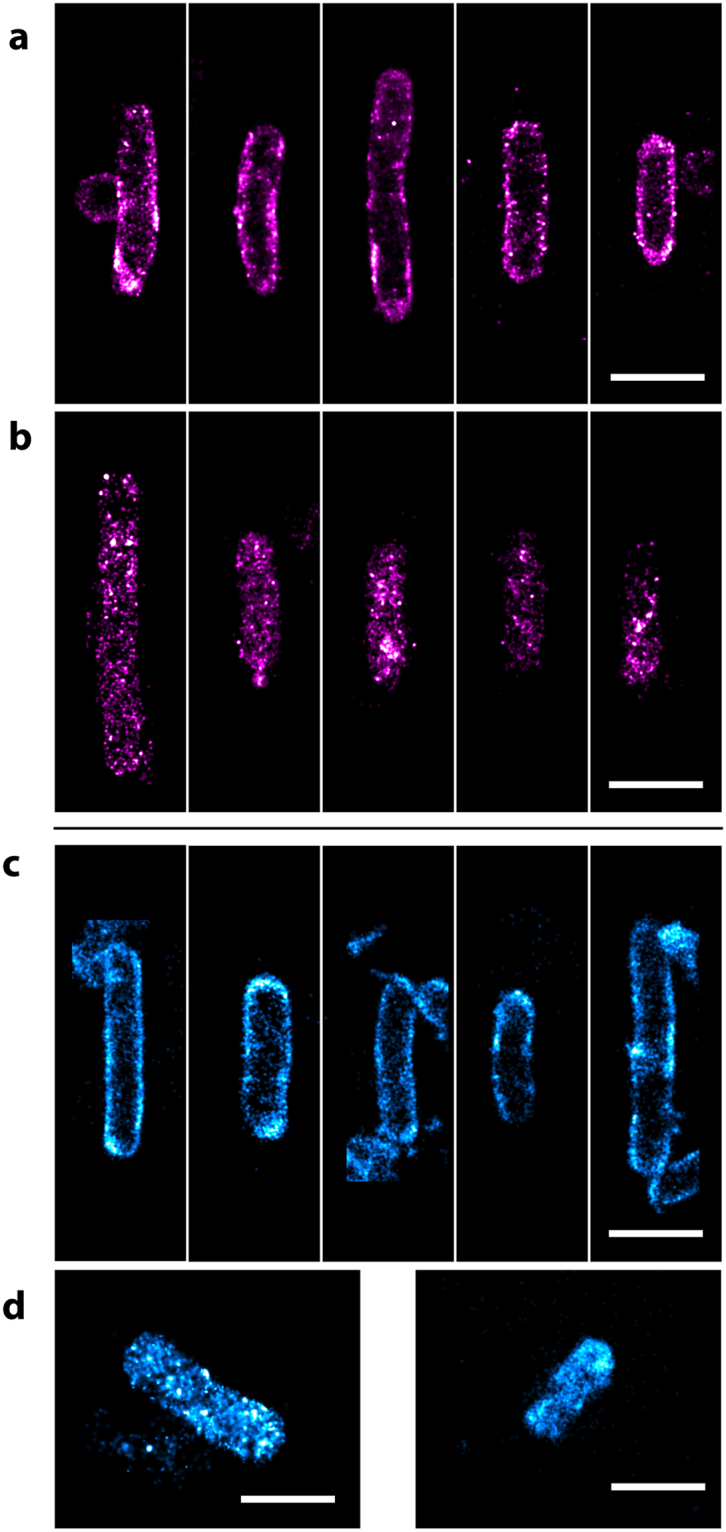
STORM imaging of CpcA-PEB and CpcA-PCB in *E. coli*. (**a**) CydB-CpcA-PEB localized to the cytoplasmic membrane demonstrating a heterogenous distribution. (**b**) Reconstructions of CpcA-PEB as expressed freely into the cytoplasm showing a more even distribution. STORM imaging of (**c**) CydB-CpcA-PCB and (**d**) cytoplasmic CpcA-PCB. Images were rotated with bilinear interpolation and the scale bar represents 1 μm.

To test their utility *in vivo*, CpcA-PCB and CpcA-PEB variants were separately expressed in *E. coli*, either freely in the cytoplasm or as translational fusions to the CydB subunit of the cytochrome *bd* quinol oxidase, which is localized in the cytoplasmic membrane. Cells were harvested, washed and mounted on clean coverslips for imaging as described in the Methods section. Photo-switching behaviour was observed for both bilin variants of the proteins, using 514 nm excitation. The reconstructions of the STORM images show a heterogeneous distribution of CydB-CpcA-PEB/PCB fusions in the *E. coli* cytoplasmic membrane (Fig. 3). The PEB variant of the fusion protein demonstrated 6640 ± 2330 localizations per cell (n = 8) whilst the PCB variant had 3500 ± 1580 localizations per cell (n = 6) In contrast to the heterogeneous membrane distribution of CydB-CpcA-PEB/PCB, free CpcA-PEB/PCB showed a uniform distribution in the cytoplasm, as expected.

### *In vivo* SIM imaging

**Figure 4:**
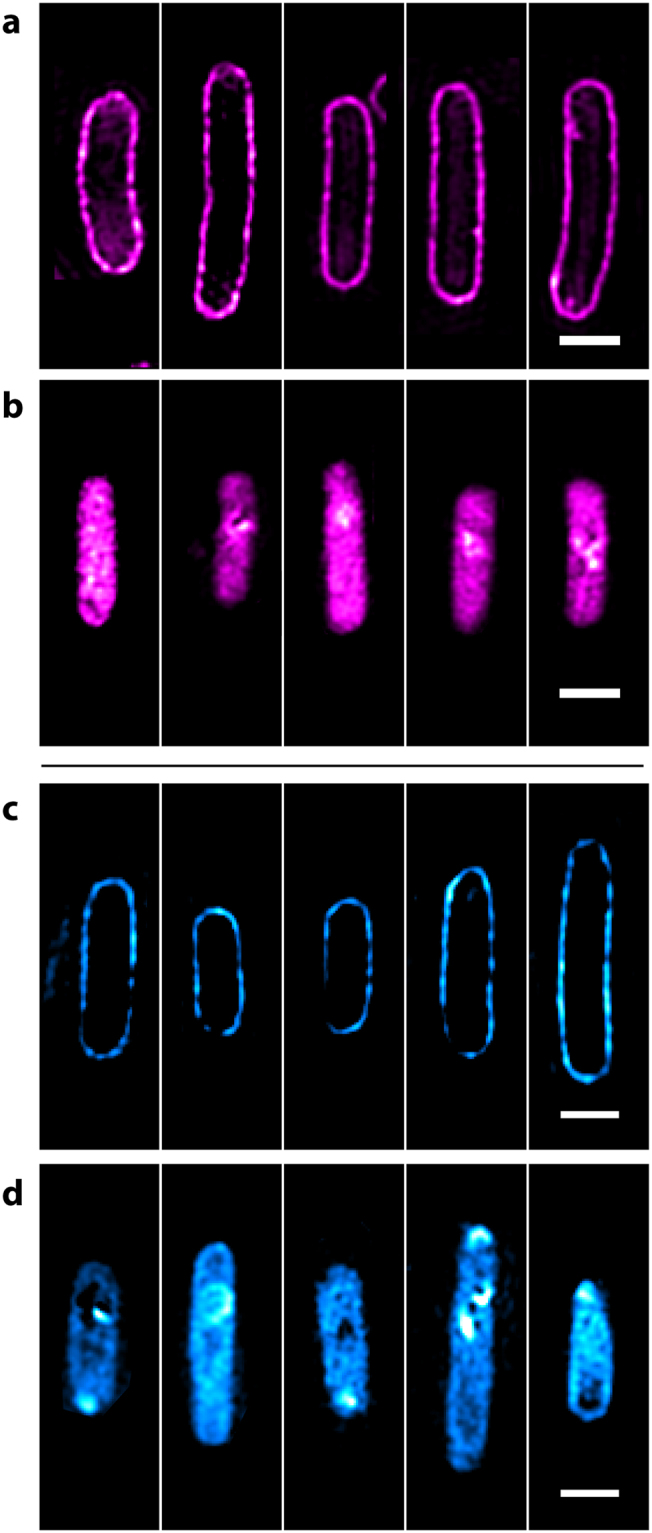
Structured Illumination Microscopy (SIM) of CpcA-PEB and CpcA-PCB in *E. coli*. (**a**) SIM imaging of CydB-CpcA-PEB localized to the cytoplasmic membrane. (**b**) Reconstructions of free CpcA-PEB in the cytoplasm. SIM imaging of **(c)** CydB-CpcA-PCB and (**d**) cytoplasmic CpcA-PCB. Images were rotated with bilinear interpolation and the scale bar represents 1 μm.

3D-SIM imaging was also conducted on the CydB-CpcA and CpcA strains and 2D reconstructions are presented in Fig. 4. Image stacks were analysed with the SIMcheck plugin to quantify fluorophore performance^16^. CpcA-PEB was particularly susceptible to photobleaching from 3D-SIM with cytoplasmically located ‘free’ protein undergoing an (89.3 ± 2.4)% intensity variation and a mean decay of (61.4 ± 2.2)% over the course of 135 images (number of cells = 14). This suggests that PEB is not suitable for 3D SIM, but it was suitably emissive for 2D reconstructions. CpcA-PCB was much more robust to imaging (Supplementary Figure S5), especially when located in the cytoplasm where it exhibited very little evidence of photobleaching with an intensity variation (28.2 ± 8.6)% and a mean decay of (18.4 ± 9.8)% over the course of 195 images (number of cells = 16).

## Discussion

We have demonstrated the application of two novel protein-ligand labels, CpcA-PEB and CpcA-PCB, for localization microscopy both *in vitro* using nanopatterning and *in vivo* in *E. coli*, where it was used to label a membrane protein complex. In addition, we have demonstrated the feasibility of using CpcA-PEB and CpcA-PCB as fluorescent labels for 2D-SIM and 3D-SIM imaging, respectively. The differences in behaviour of the protein with these two imaging modalities arises from the difference in illumination intensity, whereby CpcA exhibits continuous fluorescence at lower illumination intensities and photoswitching at higher illumination intensities. The CpcA protein is derived from the light-harvesting antenna, the phycobilisome, of the cyanobacterium *Synechocystis* sp. PCC 6803, and binds its weakly absorbing and fluorescing linear tetrapyrrole (bilin) ligands within a sterically defined site, which transforms these pigments into highly coloured and strongly fluorescent chromophores^3^. Free bilin chromophores, as well as those of denatured proteins, adopt a cyclo-helical conformation that is only very weakly fluorescent^17,18^. Thus, there is almost no background signal from the apoprotein or unattached bilins (Supplementary Figure S6), and the only localization signals observed are from the CpcA-bound form of the pigment.

Transient absorption studies of the triplet yield (Fig. 1) show the basis for the photostability of CpcA-PCB in particular (Supplementary Figure S5), which arises from nearly complete decay of the lowest singlet excited state to the ground state, with virtually no formation of long lived triplet states that could produce reactive oxygen species. The brightness values for CpcA-PCB and CpcA-PEB, 23 mM^−1^cm^−1^ and 75 mM^−1^cm^−1^ respectively, lie within the range of most fluorescent proteins that emit maximally up to 600 nm. For fluorescent proteins in the 630–720 emission range and classified as far-red and near-infrared, the low quantum yield of fluorescence lowers the brightness; only mKate2 (emission maximum 633 nm; brightness 25^19^) compares with CpcA-PCB (emission maximum 644 nm; brightness 23) and whilst a photoactivatable variant known as PAmKate has been developed, it comes with the cost of reduced brightness (emission maximum at 628 nm; brightness 8.6)^20^. In the present study, we show that both CpcA-PCB and CpcA-PEB can be produced as translational fusions to a membrane protein, and bilin ligation proceeds as for the free CpcA protein. The membrane distribution of the labelled target, in this case the CydB subunit of the cytochrome *bd* quinol oxidase, was imaged by STORM. A previous PALM imaging study used CydB labelled with mMaple, revealing clusters of localizations on 50–200 nm length scales^21^. This work was conducted with the tagged CydB produced from the native promoter, whereas here we used a plasmid-based over-expression system that probably results in much higher levels of CydB being inserted into the membrane. Thus we cannot draw conclusions about the localization of the *bd* quinol oxidase from our data; however, the purpose of our study was to demonstrate the applicability of CpcA-PCB and CpcA-PEB for super-resolution imaging *in vivo* rather than to gain any biologically relevant insight.

Given the wide variety of bilins in nature, the ability to engineer new bilin biosynthetic pathways^3,17^, and the potential for spectral tuning by reconfiguring their binding sites within CpcA, these pigment proteins have great potential for super-resolution imaging. Additionally, because CpcA does not exhibit photochromic switching, it does not occupy two spectral windows like EosFP^22^, which more easily facilitates multicolour imaging.

Recent developments offer further possibilities for this group of chromophoric proteins. The smURFP proteins incorporate PCB into the α-subunit (ApcA) of allophycocyanin of the cyanobacterium *Trichodesmium erythraeum*, and the extinction coefficient and quantum yield make this homodimeric FP suitable for intracellular imaging^5^. Further spectral expansion of biliproteins for imaging is made possible by the recent discovery of cyanobacteria that can grow in infrared light between 700 and 800 nm^23–25^. These bacteria synthesise a red-shifted chlorophyll, Chl *f*, and they also modify their phycobiliproteins accordingly^23^. Recombinant expression in *E. coli* of variants of the ApcE2 protein from *Synechococcus* sp. PCC 7335 yields phycobiliproteins with absorption maxima at 711 nm and emission maxima at 726 nm^26,27^, providing more scope for varying phycobiliprotein-based LM. Recently, thermophilic, near-infrared phycobiliproteins have been reported that are suitable for superresolution imaging^28^. Whilst mammalian expression of CpcA has not been demonstrated here, the smURFP biliprotein has been expressed in the HEK293A mammalian cell line^5^ and this, combined with the prior presence of the haem oxygenase enzyme^29^ and successful expression of PCB^30^, indicates that CpcA could be suitable for mammalian imaging.

In this work, we show that phycobiliproteins also have potential, not only for conventional fluorescence microscopy, but also for super-resolution imaging. In particular, we have demonstrated that CpcA-PEB, with a higher emissive yield and number of events, is more suitable than CpcA-PCB for STORM. However, CpcA-PCB is more resistant to bleaching, a key prerequisite for fluorophores used in SIM. The 18 kDa CpcA protein is relatively small compared with 27 kDa for the GFP family for example, are strongly absorbing and have a higher fluorescence quantum yield. Additionally, the emission spectra of CpcA proteins can be altered without having to create new genetic fusions, simply by altering the machinery required for a particular bilin ligand by plasmid exchange. This protein family has great potential for further such imaging applications^17^.

## Methods

### Overproduction and purification of CpcA-PCB and CpcA-PEB

*Escherichia (E.) coli* BL21(DE3) was grown with shaking (200 rpm) at 37 °C in Luria-Bertani (LB) broth with appropriate antibiotics. For growth on plates LB was supplemented with 1.5% (w/v) agar. Two-plasmid systems like those described in Alvey *et al*.^3^ were used for production of N-terminally His_6_-tagged CpcA with either PCB or PEB attached. For CpcA-PCB, *E. coli* BL21(DE3) was co-transformed with pBS414v, which contains the *Synechocystis* sp. PCC 6803 (hereafter *Synechocystis* 6803) *cpcA* gene (with a sequence encoding an N-terminal His_6_-tag, producing His_6_-CpcA) along with *cpcE* and *cpcF* that together encode a phycocyanobilin:CpcA lyase, and pPcyA, which encodes cyanobacterial heme oxygenase (Hox1) and the ferredoxin-dependent biliverdin reductase, PcyA. To produce CpcA-PEB, the pBS405v (His-CpcA) and pCOLAduet-*cpcEF-pebS-HO1* (PBP lyase, PEB synthase and Hox1) plasmids were used. For construction of the pCOLADuet-*cpcEF-pebS-HO1*, a *Kpn*I site was introduced into pCOLAduet-*cpcEF-pebS* ^3^ by using the QuikChange II XL site-directed mutagenesis kit (StrataGene) and primers ggtF and ggtR (see Table S1 for primer sequences). Restriction digestion analysis and DNA sequencing were used to verify the introduced *Kpn*I site. The resulting plasmid was digested with *Kpn*I and *Xho*I. The HO1 gene was cloned into the digested vector as a *Kpn*I-*Sal*I fragment recovered from plasmid pPcyA^3^, and the resulting plasmid, pCOLADuet-*cpcEF-pebS-HO1* was verified by DNA sequencing. Details of all plasmids and the antibiotics used to maintain them in *E. coli* are given in Table S2.

Protein production was performed at 18 °C for 16 h after induction with 0.4 mM isopropyl β-D-1-thiogalactopyranoside (IPTG) when cells had reached an optical density at 600 nm (OD_600_) of 0.6 at 37 °C. Cells were harvested by centrifugation (5000 rpm, 4 °C, 15 min), resuspended in binding buffer (50 mM Tris pH 7.4 with 500 mM NaCl and 5 mM imidazole) and broken by sonication on ice. Recombinant protein was initially purified from the resulting cell free extract by immobilised nickel affinity chromatography. Protein which remained bound after washing with binding buffer with 20 mM imidazole was eluted in 50 mM Tris pH 7.4 with 150 mM NaCl and 400 mM imidazole. In the case of PEB- and PCB- conjugated CpcA, the eluted proteins were visibly pink or blue, respectively. Proteins were further purified by ion-exchange chromatography on a HiTrap Q Sepharose column (GE Healthcare); proteins were eluted with a linear gradient of 0–1 M NaCl over 30 column volumes in 50 mM Tris pH 7.4, followed by size-exclusion chromatography on a S200 Superdex column (GE Healthcare) in 50 mM Tris-HCl pH 7.4 with 200 mM NaCl.

### Photophysical properties of purified CpcA-PCB and CpcA-PEB

Photophysical studies were performed at room temperature on dilute (µM) samples in 20 mM Tris-HCl pH 7.4 buffer. Samples used to determine the fluorescence quantum yields and singlet excited state lifetimes were purged with argon. Absorption spectra were acquired with a Shimadzu UV-1800 spectrophotometer. Static fluorescence studies utilized a Horiba Nanolog spectrofluorimeter with 2–4 nm bandpass in the excitation and detection legs; spectra were corrected for the instrument response. Fluorescence quantum yields were measured via absolute studies using a Horiba Quanta Phi integrating sphere and relative to tetraphenyl porphyrin in non-degassed toluene (Φ_f_ = 0.07^31^) and relative to a boron dipyrrin dye (*N,N*′-difluoroboryl-5-mesityldipyrrin) in degassed toluene (Φ_f_ = 0.93 found previously from a relative measurement^32^ and here using an integrating sphere); the results of the three measurements were averaged. Singlet excited state lifetimes were measured by two techniques: (1) fluorescence decay using time correlated single photon counting (TCSPC) using a Becker & Hickl Simple Tau detection system (with an instrument response of 200 ps) and 100-fs excitation flashes from a Spectra Physics Mai-Tai Ti:Sapphire laser and pulse picker operating at 1 MHz, and (2) transient absorption spectroscopy that utilized an Ultrafast Systems Helios spectrometer and a Spectra-Physics amplified femtosecond laser system that delivered 100 fs pump and probe flashes at 1 kHz; the results of the two measurements were averaged. The transient absorption studies also allowed measurement of the yield of the intersystem crossing from the singlet to triplet excited state (the triplet yield). The latter value together with the fluorescence quantum yield afforded the yield of internal conversion of the singlet excited state to the ground state (Φ_ic_ = 1 − Φ_f_ − Φ_isc_).

### Nanopatterning

CpcA nanopatterns were created on glass coverslips using polystyrene masks and self-assembled monolayers as described previously^14^. To produce a template mask, an offcut diffraction grating (303 nm on/ 303 nm off) purchased from LightSmyth technologies was cleaned with piranha solution (3:1 H_2_SO_4_:H_2_O_2_) and spin-coated at 2,000 rpm with 58 mg ml^−1^ polystyrene dissolved in toluene until dry. The mask was lifted from the template by submersion in ultrapure deionized water using surface tension and placed onto a piranha-cleaned silicon wafer with the channels running perpendicular to a crystallographic axis. To open the channels, the mask was dried under vacuum before the wafer was cracked along the axis, making a clean break. Masks were then transferred to piranha-cleaned coverslips and dried under vacuum to remove any remaining water. For the two separate protein variants, different processes were used to create the nanopatterns. For CpcA-PCB, a self-assembled monolayer was created in the channels by vapor deposition by placing the coverslips under an anhydrous nitrogen atmosphere with 35 µl (3-mercaptopropyl)trimethoxysilane (MPTMS) (Sigma-Aldrich) before being placed into vacuum. Once the monolayer had formed the masks were removed and the coverslips submerged in 1 µl ml^−1^ 2[methoxy(polyethyleneoxy)6–9propyl]trichlorosilane in toluene for 90 min to coat the exposed glass, reducing non-specific binding of the protein to the negatively charged surface. Alternatively, for CpcA-PEB 1 H,1 H,2 H,2H-perfluoro-octyltriethoxysilane (20 µl) was vapor deposited under vacuum from an anhydrous nitrogen atmosphere. This created a negative of the template which was then removed. The exposed non-fluorinated substrate was incubated in 15 mM MPTMS dissolved in toluene to create the nanopattern. For both patterns, succinimidyl 4-(N-maleimidomethyl)cyclohexane-1-carboxylate (SMCC) was dissolved in dry dimethyl sulfoxide to form a 1 mM solution which was used as a linker to bind the MPTMS. CpcA was buffer-exchanged into phosphate buffered saline (PBS) using a PD10 desalting column (GE Healthcare) because Tris-HCl can compete for the SMCC linker. CpcA (1 µM) in PBS was incubated on the surface and allowed to bind to the SMCC-MPTMS pattern. PBS was used to wash the coverslip, which was then sealed onto a glass slide.

### Imaging of CpcA nanopatterns

All STORM imaging was performed on a Nikon TiE inverted microscope equipped with a Nikon Apo TIRF 60× oil objective (N.A. 1.49), and all lasers were free-space-coupled into the back port of the microscope. The Nikon Perfect Focus System was used to maintain the axial position throughout the imaging period. Monitoring of lateral drift was performed with either 0.1 µm 540/560 FluoSpheres (ThermoFisher) as fiducial markers or through cross-correlation^33^. Reconstructions were performed with the ThunderSTORM^17^ software package using the wavelet filter and Maximum Likelihood Estimator fitting. CpcA-PCB nanopatterns were imaged with a 514 nm Coherent Sapphire laser at a power density of 1.6 kW/cm^2^. Imaging was performed with a ZT514/647rpc-UF2 dichroic beamsplitter (Chroma Technology) and a ZET532/640 M emission filter (Chroma Technology). 10,000 frames were collected with a 40 ms exposure on a Zyla 4.2 sCMOS camera (Andor Technology) with an effective pixel size of 108.3 nm. The camera was controlled with the native Andor Solis software. CpcA-PEB nanopatterns were imaged with a 532 nm Coherent Sapphire laser in wide-field at a power density of 1 kW/cm^2^. The laser was reflected of a 552 nm dichroic (Semrock) to excite the sample and the emission was collected through a 580/60 emission filter (Semrock). 14,000 frames were collected with a 40 ms exposure on an ORCA-Flash 4.0 V2 sCMOS camera (Hamamatsu Photonics) controlled by the native HCImage Live software. The frames were processed with ThunderSTORM^15^.

### Photoswitching properties of CpcA-PCB and CpcA-PEB

An MPTMS monolayer was created by incubating piranha cleaned coverslips in a 1:10,000 MPTMS/ethanol solution. Coverslips were then washed with ethanol and dried before incubation with 1 mM SMCC dissolved in dimethyl sulfoxide for 20 minutes. A 10 nM CpcA solution was applied to the coverslips and incubated for 30 minutes followed by extensive washing with PBS and mounting. Imaging was performed using the same regime as the nanopatterned protein (above).

The on rate of CpcA-PCB and CpcA-PEB were found be collating the time-period between activations of single fluorophores over a 30-minute window. The data were then fit to an exponential model to obtain the on-switching rate constant. Similarly, for the off rate the duration of each emission was recorded and fit to an exponential model to obtain the off switching rate constant. The contrast ratio was obtained by taking the cross-sectional profile before, so as to get a true representation of the dark state and not the photobleached molecule, and during an event.

### Calculation of extinction coefficients for CpcA-PCB and CpcA-PEB

Calculation of the native molar extinction coefficients at the absorption maximum was carried out through comparison of the native absorption spectrum to that of the denatured protein. The extinction coefficients for CpcA-PCB/PEB has previously been reported at 663 nm and 555 nm in acidic urea as 33,200 and 43,300 respectively^34,35^. Additionally, the extinction coefficients of the minor peaks at 352 nm and 308 nm for PCB and PEB were 32,600 and 18,300 respectively. Purified CpcA was diluted in either PBS (native) or 8 M urea pH 1.9 with 10 mM β-mercaptoethanol (denatured) and the absorption spectrum measured from 300 nm to 800 nm on a Cary 60 spectrophotometer in triplicate (Supplementary Figure S7). As the concentrations are equal, the native extinction coefficient can be found ratiometrically from the absorbance. Native CpcA-PCB has an extinction coefficient of 100,265 at 624 nm whereas CpcA-PEB has an extinction coefficient of 112,590 at 556 nm. The extinction coefficients of the secondary peaks were lower in native conditions and were 17,702 at 352 nm for PCB and 14,796 at 308 nm for PEB.

### Production and visualisation of CpcA and CydB-CpcA in *E. coli*

To label the cytochrome *bd*-1 oxidase with CpcA we generated a translational fusion of the *E. coli cydB* and *Synechocystis* 6803 *cpcA* genes. CydB was chosen because its C-terminus is exposed to the cytoplasmic side of the membrane,^36^ and the protein is known to be functional when tagged with GFP^37^. The *cydB* and *cpcA* genes were amplified from *E. coli* or *Synechocystis* 6803 genomic DNA by PCR with Q5 High-Fidelity DNA Polymerase (New England Biolabs Inc.) and primer pairs oligo_AH330/oligo_AH331 or oligo_AH332/oligo_AH333 respectively (Table S1). The two PCR products were joined by overlap extension PCR with primer pair oligo_AH330/oligo_AH333, generating a product in which the two genes were separated by 15 bp of sequence encoding a five amino acid linker as in^38^. This fragment was digested with *Nco*I and *Eco*RI and cloned into the same sites of the pBS414v and pBS405v plasmids, generating plasmids pAH171 and pAH173 in which the *cydB*-*cpcA* fusion gene replaced His-*cpcA*. Co-transformation of *E. coli* with pAH171 and pPcyA or pAH173 and pCOLAduet-*cpcEF*-*pebS*-*HO1* results in production of CydB-CpcA-PCB and CydB-CpcA-PEB, respectively. For imaging, cells were grown as described above using the appropriate plasmid combinations and antibiotics (Table S2). Cells were harvested by centrifugation (5000 rpm, 4 °C, 15 min) and cell pellets were washed in 25 mM HEPES (4-(2-hydroxyethyl)-1-piperazineethanesulfonic acid), pH 7.5.

For STORM, cells were resuspended in PBS and incubated on piranha-cleaned coverslips coated with poly-L-lysine (0.1%) and a dilute solution of 540/560 0.1 µm Fluospheres for drift correction (ThermoFisher Scientific). Cells were imaged with a 514 nm Coherent Sapphire laser at a power density of 1.6 kW/cm^2^. Imaging was performed with a ZT514/647rpc-UF2 dichroic beamsplitter (Chroma Technology) and a ZET532/640 M emission filter (Chroma Technology). 10,000 frames were collected with a 40 ms exposure on a Zyla 4.2 sCMOS camera (Andor Technology).

For SIM, cells were resuspended in PBS and incubated on piranha-cleaned coverslips coated with poly-L-lysine (0.1%). Samples were mounted in SlowFade Diamond Antifade (ThermoFisher Scientific) and imaged in the Wolfson Light Microscopy Facility at the University of Sheffield. Samples were imaged on a DeltaVision OMX V4 microscope (GE Healthcare) with the Blaze 3D-SIM module equipped with a 60× 1.42 NA oil objective. CpcA-PEB was imaged with a 514-nm laser with emission collected through a 609/37 bandpass filter. CpcA-PCB was imaged with a 642 nm laser with emission collected through a 683/40 bandpass filter. Axial sectioning was achieved with a step size of 125 nm and the resultant image stacks were reconstructed with the SoftWoRx 6.0 software (GE Healthcare).

### Data availability

All data that support the findings of this study are available from the corresponding author upon request.

## Electronic supplementary material

Supplementary Information
