# Supplementary material for: Repurposing a photosynthetic antenna protein as a super-resolution microscopy label
Source: Sci Rep. 2017 Dec 1;7:16807. doi: 10.1038/s41598-017-16834-z (PMC5711914; doi:10.1038/s41598-017-16834-z)
Supplement: Supplementary file 1 — Supplementary Information [file 41598_2017_16834_MOESM1_ESM.pdf]

Repurposing a photosynthetic antenna protein as a super-resolution microscopy label

Samuel F. H. Barnett[1], Andrew Hitchcock[1], Amit K. Mandal[2], Cvetelin Vasilev[1], Jonathan M. Yuen[2], James Morby[1], Amanda A. Brindley[1], Dariusz M. Niedzwiedzki[2], Donald A. Bryant[3], Ashley J. Cadby[4], Dewey Holten[2] & C. Neil Hunter[1]

[1]Department of Molecular Biology and Biotechnology, Firth Court, Western Bank, Sheffield S10 2TN, UK.

[2]Department of Chemistry, Washington University in St. Louis, St. Louis, MO 63130, USA.

[3]Department of Biochemistry and Molecular Biology, The Pennsylvania State University, University Park, Pennsylvania 16802, USA.

[4]Department of Physics and Astronomy, Hicks Building, Hounsfield Road, Sheffield

S3 7RH, UK.

Correspondence should be addressed to C.N.H. (c.n.hunter@sheffield.ac.uk).

SUPPLEMENTARY INFORMATION

Supplementary Figures

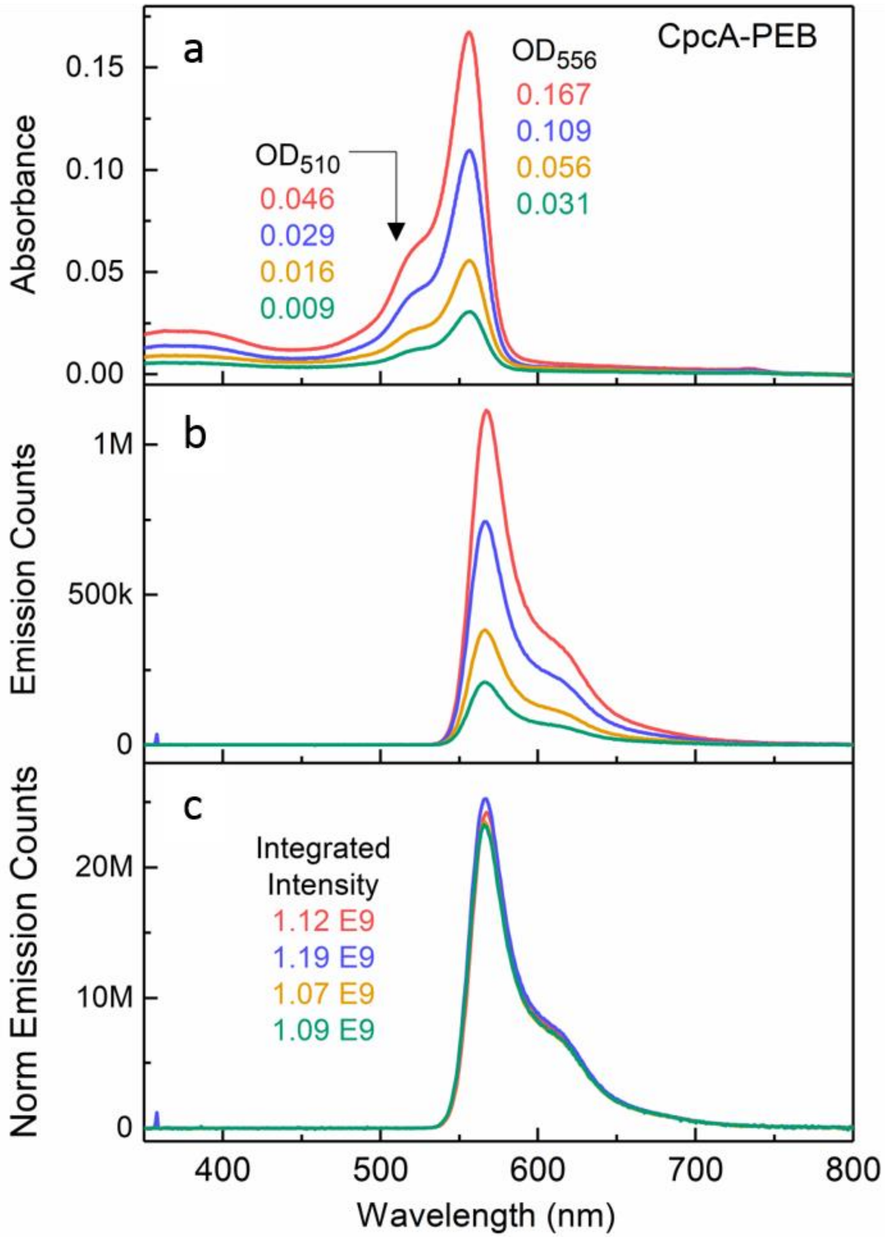

Figure S1: Fluorescence spectra vs concentration. (a) Absorption spectra of CpcA-PEB at four different concentrations of samples having the indicated OD at the peak (556 nm) and at the excitation wavelength used to acquire fluorescence spectra (510 nm). (b) Fluorescence spectra of four samples. (c) Fluorescence spectra of the four samples with intensities divided by the absorbance at the 510 nm excitation wavelength, along with the integrated (500–800 nm) intensities.

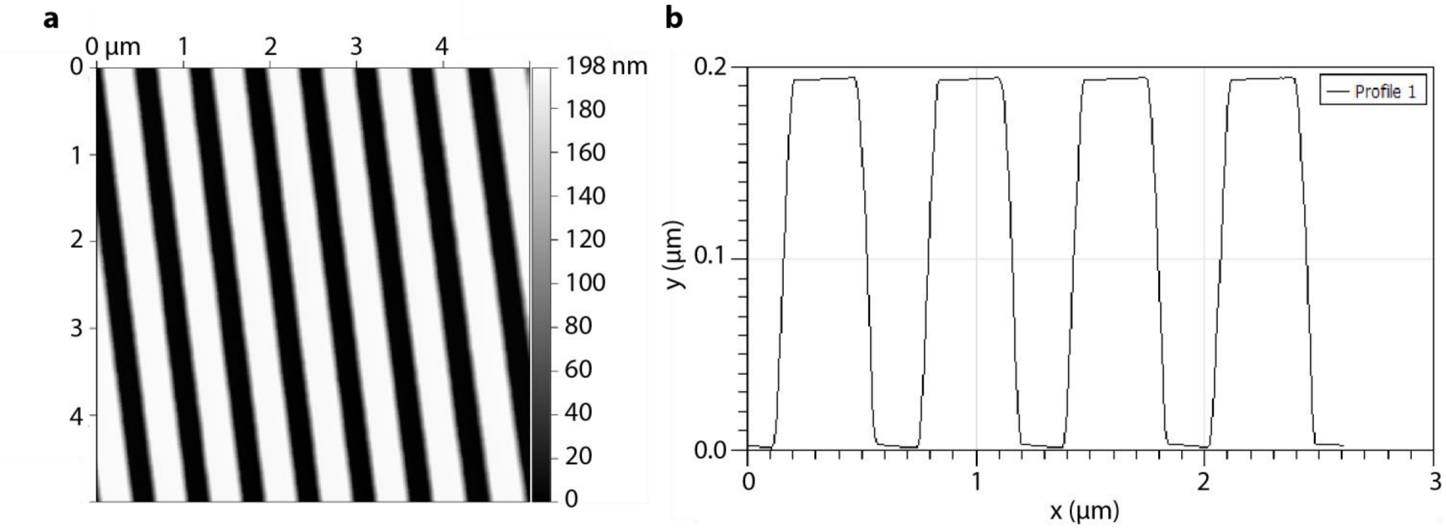

Figure S2. AFM nanopattern templates. Silicon master template imaged by AFM **(a)** demonstrate the dimensions of the pattern to be 300 nm on, 300 nm off **(b)**.

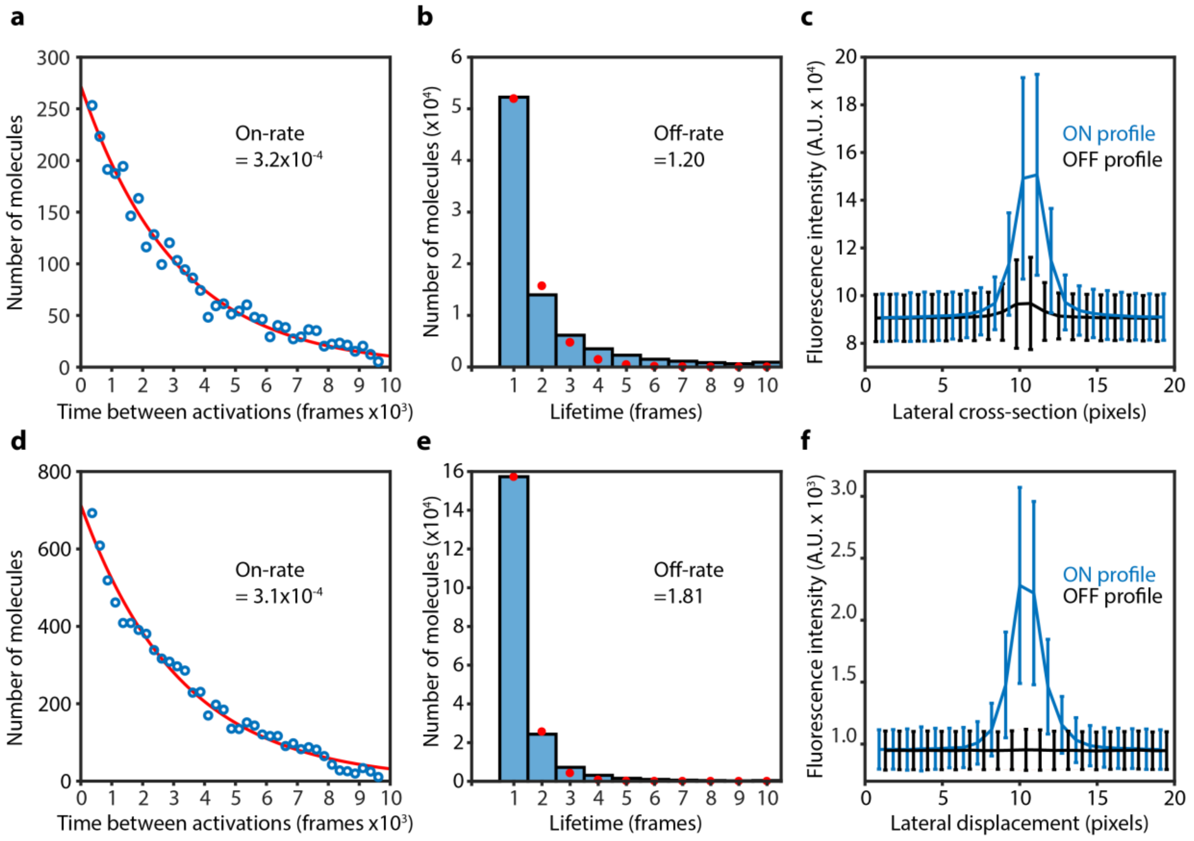

Figure S3: Photoswitching characteristics of CpcA-PCB (a-c) and CpcA-PEB (d-f). The on- (a and d) and off- (b and e) rates of the fluorophores were calculated by fitting a single order exponential functions to the period between single molecule activations and length of activations respectively. The contrast ratio (c and f) was found by the mean cross-section profile of individual events before and during emission

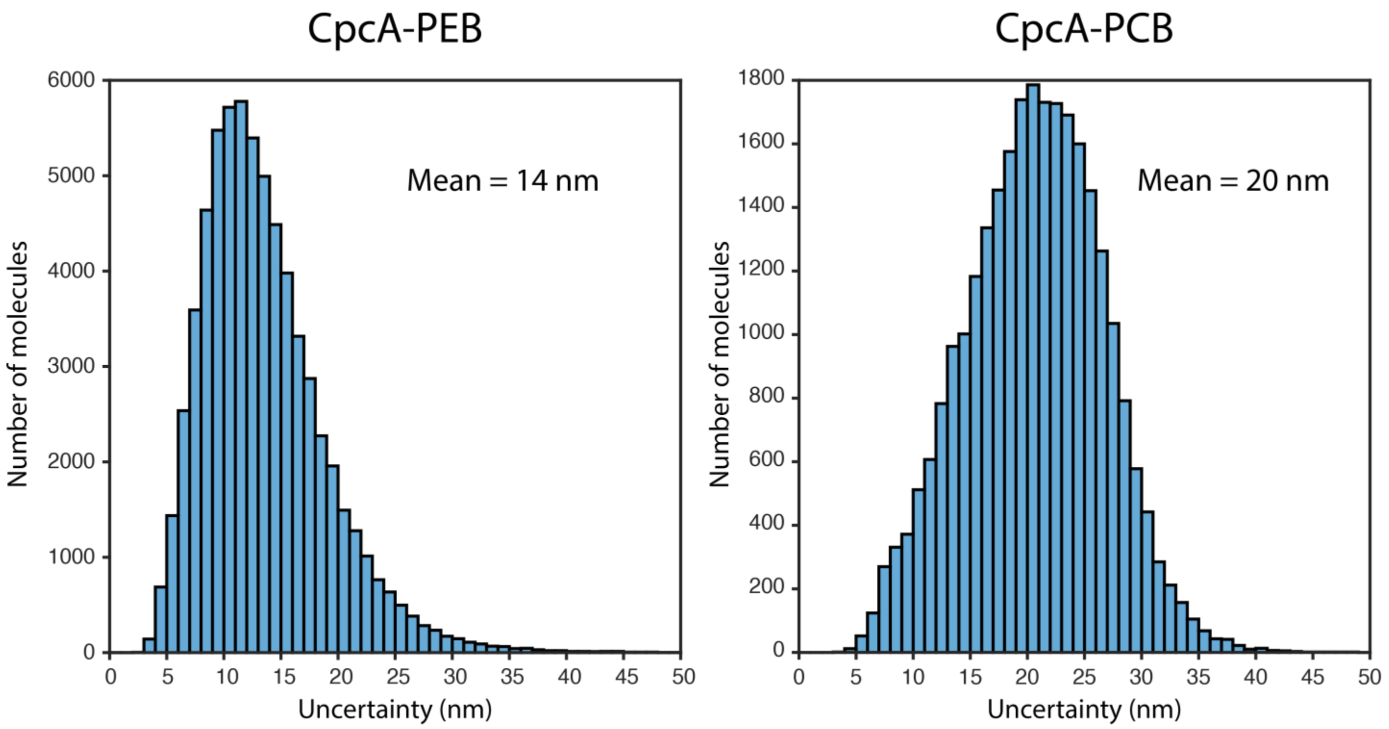

Figure S4: Calculated uncertainties of photoswitching CpcA proteins in the nanopattern datasets. Uncertainty was calculated during the reconstruction process by the ThunderSTORM plugin for ImageJ.

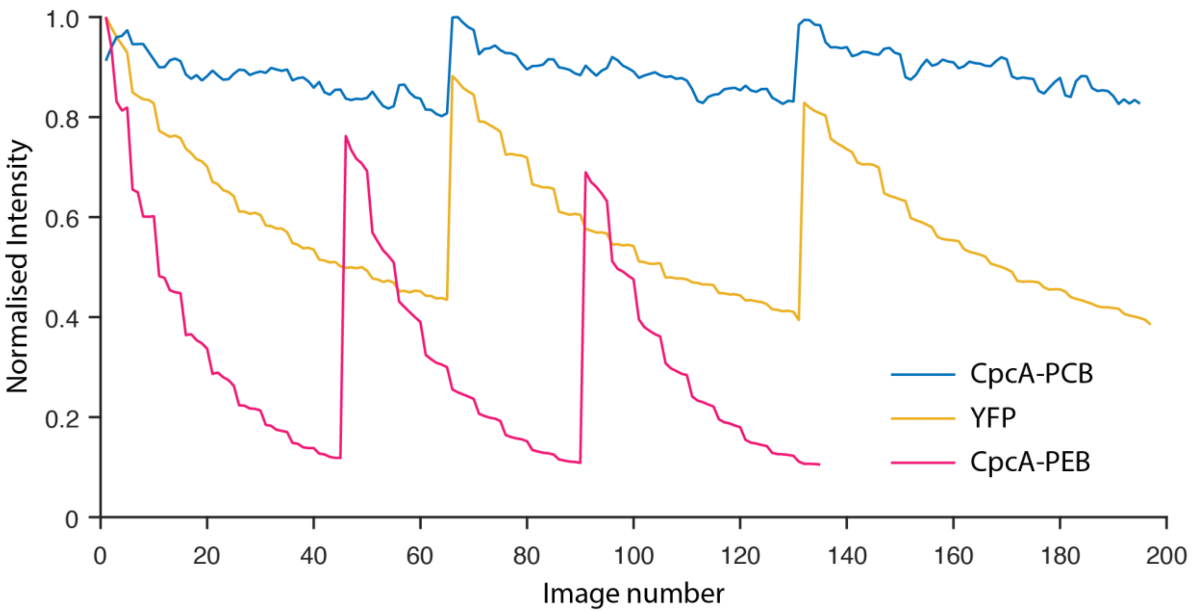

Figure S5. Robustness of CpcA to photobleaching. Fluorescence intensity decay curves of CpcA-PCB (blue), CpcA-PEB (pink) and sYFP2 (orange) under a structured illumination microscopy imaging regime.

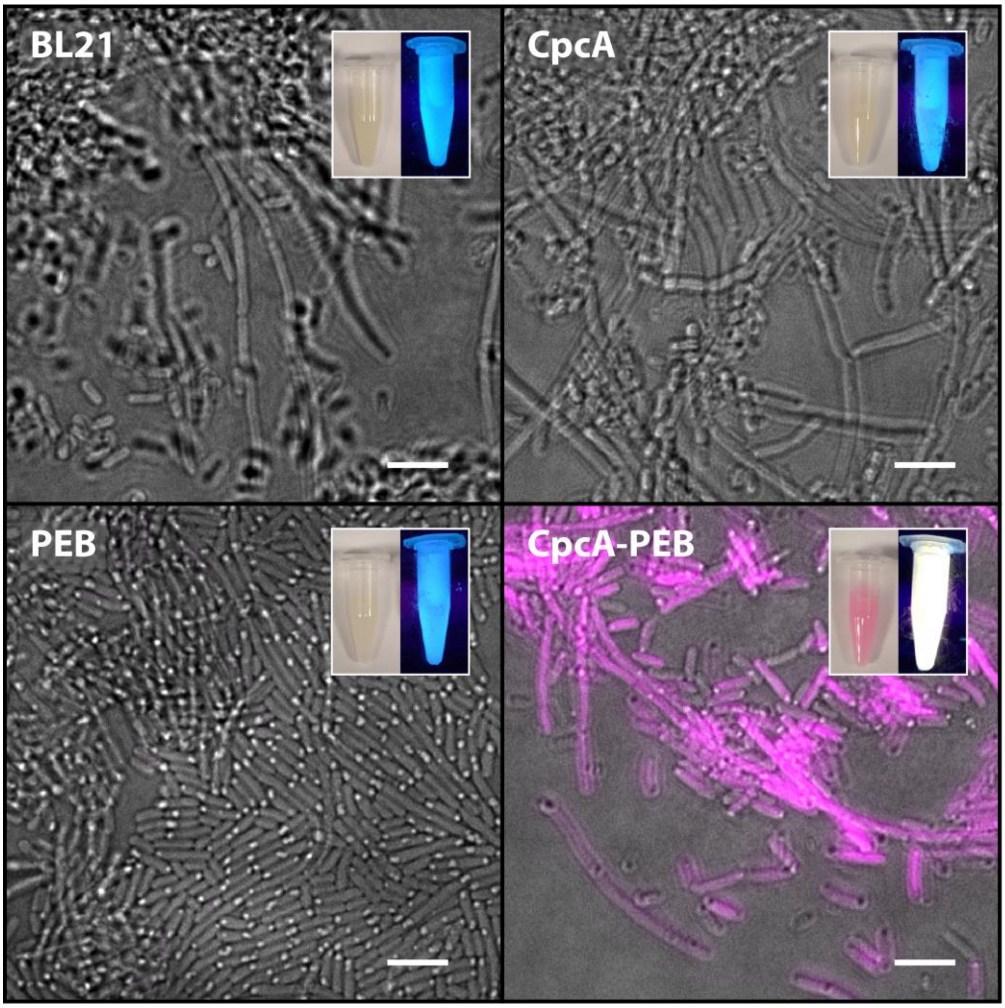

Figure S6. Comparison of *E. coli* producing CpcA, PEB and CpcA-PEB. Overlaid DIC and epifluorescence images of *E. coli* producing neither the bilin or protein component (top-left), only the CpcA apoprotein (top-right), only phycoerythrobilin (PEB) (bottom-left), or CpcA and PEB (bottom-right). All images are displayed on the same intensity scale and the scale bar represents 5 µm. The insets to each panel are the *E. coli* cell cultures of their respective panel imaged under white light (left) and UV light (right).

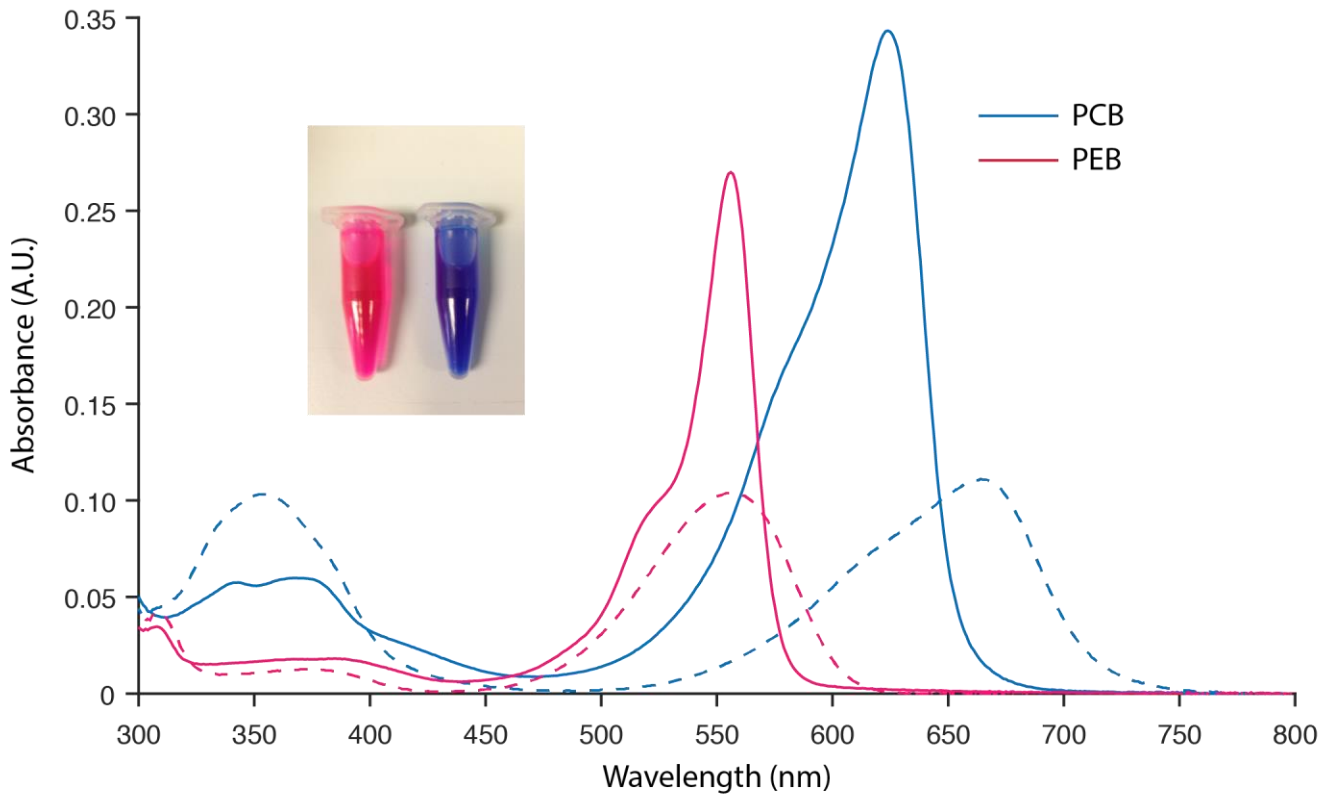

Figure S7. Calculation of chromophore extinction coefficients. The absorption spectra of native (solid lines) and denatured (dashed lines) PCB-conjugated (blue) and PEB-conjugated (pink) CpcA used to calculate the molar extinction coefficients. The displayed spectra are the average of 3 replicate traces and were normalised to zero at 790 nm. The inset panel shows the intensely coloured purified protein preparations.

Supplementary Tables

Table S1. Primers used in this study.

Name	Sequence 5'-3'	Details
oligo_AH_330	TTTCG**CCATGG**GGATCGATTATGAAGTATTGC	*cydB* forward primer, *Nco*I site in bold.
oligo_AH_331	*CATGCGACCACACAGGCCGTA*CAGAGAGTGGGTGTTACG	*cydB* reverse primer, overlap region italicised.
oligo_AH_332	*TACGGCCTGTGTGGTCGCATG*AAAACCCCTTTAACTGAAGCC	*cpcA* forward primer, overlap region italicised.
oligo_AH_333	CTATA**GAATTC**GAAGACTAGCTCAGAGCATTGATGG	*cpcA* reverse primer, *Eco*R1 site in bold.
ggtF	CGCAGCTTAATTA**GGTACC**TAGGCTGCTGC	Quikchange primers for *Kpn*I site (bold)
ggtR	GCAGCAGCCTA**GGTACC**TAATTAAGCTGCG	introduction in pCOLAduet-*cpcEF-pebS*.

Table S2. Plasmids used in this study.

Name	Proteins produced	Parent Vector	Antibiotic selection[b]	Reference
pPcyA	HOI, His_6-PcyA	pACYC Duet	Cm, 34 µg ml^{-1}	[1, 2]
pCOLAduet-*cpcEF-pebS*	CpcE, CpcF, PebS	pCOLAduet-1	Km, 50 µg ml^{-1}	[2]
pCOLADuet-*cpcEF-pebS-HO1*	CpcE, CpcF, PebS, HOI	pCOLAduet-1	Km, 50 µg ml^{-1}	This study
pBS405v	His_6-CpcA	pBS350v	Sp, 50 µg ml^{-1}	[3]
pBS414v	His_6-CpcA, CpcE, CpcF	pBS350v	Sp, 50 µg ml^{-1}	[3]
pAH171	CydB-CpcA, CpcE, CpcF	pBS414v	Sp, 50 µg ml^{-1}	This study
pAH173	CydB-CpcA	pBS405v	Sp, 50 µg ml^{-1}	This study

[a] HT: His_6-tagged. [b] Antibiotic concentration used to select for the plasmid: Cm: chloramphenicol; Km: kanamycin; Sp: spectinomycin.

Supplementary Referernces

1. Biswas, A. et al. Biosynthesis of cyanobacterial phycobiliproteins in Escherichia coli: chromophorylation efficiency and specificity of all bilin lyases from Synechococcus sp. strain PCC 7002. Applied and Environmental Microbiology 76, 2729–2739 (2010).

2. Alvey, R. M., Biswas, A., Schluchter, W. M. & Bryant, D. A. Attachment of noncognate chromophores to CpcA of Synechocystis sp. PCC 6803 and Synechococcus sp. PCC 7002 by heterologous expression in Escherichia coli. *Biochemistry* **50,** 4890–4902 (2011).

3. Tooley, A. J., Cai, Y. A. & Glazer, A. N. Biosynthesis of a fluorescent cyanobacterial C-phycocyanin holo-alpha subunit in a heterologous host. *Proc. Natl. Acad. Sci. U.S.A.* **98,** 10560–5 (2001).
